# Working from Home in Urban China during the COVID-19 Pandemic: Assemblages of Work-Family Interference

**DOI:** 10.1177/09500170221080870

**Published:** 2022-06-02

**Authors:** Li Sun, Tao Liu, Weiquan Wang

**Affiliations:** University of Leeds, UK; Zhejiang University, China; East China University of Political Science and Law, China

**Keywords:** assemblages, boundary theory, COVID-19, urban China, work-family interference, working from home

## Abstract

During the COVID-19 pandemic, millions of workers globally have been forced to work from home. Empirical data from Chinese cities in the Hubei province reveal work productivity decreased among many respondents working from home in 2020, primarily due to family interference with work. Such interference stems not only from the domain of daily life but also from other family members’ e-working and e-learning. Conversely, respondents’ work interferes with family; thus, interference operates bi-directionally. This article proposes an analytical framework of work-family interference along three dimensions: work-daily life, work-work, work-study, and each dimension can be understood through four distinct aspects: temporality, physicality, vocality, digitality. Remote workers encounter ‘assemblages of work-family interference’, consisting of a heterogeneous mixture of these dimensions and aspects. Furthermore, some factors (e.g., living patterns, work culture, digital infrastructure) constrain effective work-family boundary management among urban households.

## Introduction

During the COVID-19 pandemic, many governments across the world encouraged office workers to work from home as a measure to contain the spread of the virus. According to the International Labour Organization (ILO), around 68% of the world’s total workforce live in countries with recommended or required workplace closures. Work from home is referred to as ‘a working arrangement in which a worker fulfils the essential responsibilities of his/her job while remaining at home, using information and communications technology (ICT)’ (ILO, 2020: 5). Scholars also use the concepts of teleworking, telecommuting, remote working, or e-working to depict alternative working arrangements outside of the workplace (Allen et al., 2015; Grant et al., 2013; Haddon and Silverstone, 1995; Kirk and Belovics, 2006; Nilles, 1975). This article mainly uses the term ‘work from home’ (hereafter WFH) and individuals who work from home are referred to as ‘remote workers’.

With the unprecedented sudden changes in working arrangements in 2020, COVID-19 has triggered massive experiments on WFH globally. COVID-19 provides a unique context to study WFH, as some of its features during the pandemic are significantly different from pre-COVID conditions. For example, WFH during COVID-19 has been mandated by governments (Walker et al., 2020), in contrast to a voluntary decision pre-COVID (Versey, 2015). Moreover, there has been much less time to plan and prepare for WFH during COVID-19 compared to pre-COVID times. Finally, anxiety and stress levels have substantively increased during COVID-19 (Usher et al., 2020).

Despite the recent increased media attention on the issue of WFH (BBC, 2020; CNN, 2020), there is an acute need for more empirical research to provide an up-to-date understanding of this issue; only a handful of empirical studies have been published to examine WFH during COVID-19 (Allen et al., 2021; Craig and Churchill, 2021; Feng and Savani, 2020; Waizenegger et al., 2020). China was the first country to impose restrictive measures to contain the spread of COVID-19, such as lockdowns, curfews, working from home, online schooling, travel bans, etc. (Huang et al., 2020). Hubei province, known as the first epicentre of COVID-19, was locked down at the end of January 2020 (Gao and Yu, 2020). On 7 February, the central government addressed the necessity of working from home nationally (Govcn, 2020a), which provoked the world’s biggest WFH experiment; around 400 million Chinese have engaged in WFH since February 2020 (Mobtech, 2020), compared to only 4.9 million people in China working from home in 2018 (Center for International Knowledge on Development [CIKD], 2020).

In this research, empirical data were collected in China’s Hubei province to explore the phenomenon of WFH during the COVID-19 pandemic. As work-family conflicts represent one of the core debates in the existing literature on WFH (Grant et al., 2013; Koslowski et al., 2019), the central research question concerns remote workers’ experiences with work-family interference while working from home during COVID-19. Existing analytical approaches to WFH do not portray the full picture of work-family interference among remote workers, because they largely address only the work-daily life dimension (e.g., housework, childcare) (Amstad et al., 2011; Derks et al., 2016); other types of interference are overlooked. Because family members’ working and studying arrangements have also shifted online during the pandemic, work-family interference stems not only from the domain of daily life but also from adult family members’ e-working and children’s online learning.

In this article, an analytical framework of ‘assemblages of work-family interference’ is proposed to explore work-family interference along three dimensions (i.e., work-daily life, work-work, work-study). Firstly, work-daily life interference refers to the interference between work and daily life (e.g., housework, childcare, leisure), such as when a Zoom meeting running over into the evening may interfere with dinner plans. Secondly, work-work interference involves the interference between an individual’s work and other family members’ work, for example, when a dual-career couple both work from home. Crowded home workspace may cause interruptions to each other’s work. Thirdly, work-study interference concerns the interference between work and children’s e-learning at home. During the lockdown, online learning was carried out at home for students, which required extra input and assistance from parents. For example, children may need to use their parents’ computers for e-learning, which interferes with remote work schedules. Through empirical research, this article not only provides a comprehensive understanding of work-family interference while working from home during a global crisis like COVID-19 but its proposed framework of ‘assemblages of work-family interference’ also advances conceptual debates in the field of WFH, providing novel insights and implications for future studies.

## Literature review

Existing research points out the paradoxical effect of working from home. On the one hand, WFH brings some benefits for workers; it frees up commuter time, avoids office distraction, increases job autonomy, permits flexibility in working time, and enhances work-life fusion (e.g., combining family and work demands) (Haeger and Lingham, 2014; Ter Hoeven and Van Zoonen, 2015). Hence WFH boosts job productivity and well-being satisfaction (Fonner and Stache, 2012). On the other hand, some researchers argue WFH has several negative consequences, including feelings of social and professional isolation, distractions from the home environment (Allen et al., 2015), an ‘always-on’ culture resulting in work intensification (Derks et al., 2016; Mullan and Wajcman, 2019), and work-family inter-role conflict (Amstad et al., 2011; Haddon and Silverstone, 1995).

Studies on work-family fusion/conflict highlight the blurred boundaries between home and work (Koslowski et al., 2019). Boundary theory addresses the micro-level processes of role transition, for example, between work roles and family roles (Ashforth et al., 2000). Family responsibilities include childcare, yard work, cooking, cleaning, repairs, shopping, and paying the bills (Frone et al., 1992: 726). Flexibility and permeability are two key concepts in boundary theory. Flexibility refers to the ability to leave one domain for the demands of another domain, and permeability emphasises the elasticity of boundaries that permit interference (Nippert-Eng, 1996). Boundaries may be temporal, physical, emotional, cognitive, and/or relational (Allen et al., 2015). For instance, physically, designated home workspaces can help avoid family interruptions (Myrie and Daly, 2009).

Effective boundary management is vital for individuals who work from home (Kossek et al., 2006; Reissner et al., 2021), as blurred boundaries may elicit work-family inter-role conflict when role pressures from the domains of work and family become mutually incompatible (Greenhaus and Beutell, 1985: 77). Ashforth et al. (2000) propose the term ‘boundary violations’ (i.e., the role transitions violating the work-home boundary) to analyse inter-role conflict, while some scholars adopt the concept of work-home ‘interference’ (Amstad et al., 2011; Derks et al., 2016). On the one hand, work roles may interfere with family roles (Baker et al., 2007); for example, an off-hours business meeting can violate family boundaries, which reduces the quality of family life. On the other hand, family roles may interfere with work roles (Feng and Savani, 2020); for instance, childcare responsibilities distracting employees from work can violate work boundaries. A recent study exploring boundary management during COVID-19 has found that having a dedicated office space and fewer household members can boost the work-nonwork balance (Allen et al., 2021). Some scholars analyse the work-family boundary from a gender perspective, arguing that women experience a higher degree of inter-role conflict than men (Fonner and Stache, 2012; Lewis, 2009; Powell and Craig, 2015), because women devote more time to the family domain (e.g., childcare, domestic chores), which unavoidably reduces their time spent in other activities (e.g., work, leisure, sleep).

Existing studies on work-family interference have extensively addressed work-daily life interference (e.g., housework, childcare) (Amstad et al., 2011; Koslowski et al., 2019), while other dimensions of interference have been overlooked (i.e., work-work interference, work-study interference). Such interferences are understudied because during ‘normal’ times, remote workers’ work settings are largely separated from family members’ work and study settings (Feng and Savani, 2020). During the COVID-19 pandemic, the home served as a multifunctional place for living, working, studying, etc. Because all family members’ activities were carried out under one roof, remote workers encountered multidimensional forms of interference between work and family. As their family members’ working and studying arrangements have also shifted online, the related interference can no longer be ignored. Thus, in addition to the conventionally discussed work-daily life interference, this study also examines work-work interference and work-study interference.

## Research methods

In April and May of 2020, data collection was carried out in the Hubei province of China, the epicentre of the coronavirus outbreak. With a total population of 59.02 million, Hubei includes 18 administrative divisions and its provincial capital city is Wuhan. To have an overall understanding of WFH in the Hubei province during COVID-19, three cities were selected as research sites, including Wuhan, Huanggang, and Enshi, which represent different types of cities in Hubei. Wuhan, with a population of 11 million, is a major city in Hubei province. The population of Huanggang is 6.3 million, classing it as a medium-sized city. Enshi, with a population of 3.38 million, is regarded as a small city.

On 23 January 2020, lockdown was first imposed in Wuhan; soon after, all regions of Hubei province were locked down (Yang et al., 2020). In early February, a national lockdown was enforced across China. Meanwhile, a series of strict regulations (regarding curfews, travel bans, and WFH) were enacted to contain the virus. On 8 April, Wuhan was the last city to lift such regulations. This study seeks to investigate WFH among Hubei residents during this period of the lockdown (the end of January to the beginning of April 2020). In April and May 2020, data collection was conducted in the three selected cities, and mixed methods were utilised, including a quantitative survey and semi-structured qualitative interviews. Eight students at East China University of Political Science and Law assisted the authors with data collection. The three selected research sites are the hometowns of these students and they speak the local dialect. Further, these students were volunteers in their cities in response to the pandemic; the Chinese government had advocated for unprecedented levels of volunteer participation (Govcn, 2020b). To ensure student and respondent safety, an online survey and telephone interviews were employed for the data collection.

The survey was conducted via the website *Wenjuanxing* (www.wjx.cn), one of the commonly used online survey platforms in China. By setting the IP (internet protocol) function, only respondents in Wuhan, Huanggang, and Enshi could access the survey link. Hewson (2017) suggested an approach to reach satisfactory sample sizes, which involves posting the survey information on relevant social media spaces, online forums, and newsgroups. As COVID-19 volunteers in these three cities, the students advertised the survey link through their volunteer-related WeChat channels, which were viewed by local residents. WeChat, with 1.09 billion Chinese users, is the most frequently used social media application in China (Papercn, 2021). A total of 602 respondents, who worked from home during the lockdown, completed all the questions for this study. Because the online survey platform prevented respondents from continuing with subsequent questions unless all required questions on a given page were completed, these 602 questionnaires met the criteria for a valid sample size. SPSS was used for the survey analysis.

The 602 respondents included 347 in Wuhan, 153 in Huanggang, and 102 in Enshi. In the gender breakdown, 59.47% were female, and 40.53% were male. A total of 9.63% of respondents were under 20 years of age, 33.22% were 20-29 years old, 20.27% were 30-39 years old, 26.58% were 40-49 years old, and 10.31% were 50 years and above. As regards working arrangements, 49.17% of respondent households had one member working from home, and 50.83% had two or more members working from home. Regarding children’s schooling, 385 respondents, accounting for 63.95% of respondent households, had children receiving online learning and 36.05% of households had no children receiving online learning. Among these 385 respondents with children engaged in online learning, 83 had primary school children aged seven to 12, 45 had junior high school children aged 13 to 15, 83 had senior high school children aged 15 to 18, and 174 had college students or higher who were aged 19 and above. It is worth noting that due to the closure of universities and colleges during the lockdown, respondents had children living at home who were college students.

In mixed methods studies, survey findings have often been used as the basis for the selection of qualitative interviewees (Bryman, 2016: 414). After analysing the above 602 survey questionnaires, 65 respondents, diverse in gender, family structure, and the level of work-family interference, were invited to participate in semi-structured interviews via telephone. Thirty-six out of the 65 selected respondents agreed to be interviewed. Each interview lasted 30 to 60 minutes and audio recording was permitted by the interviewees. These 36 interviewees included 16 women and 20 men. Twenty-three interviewees were the only family member working from home, and 13 interviewees were part of households that had at least two members working from home. Nineteen interviewees had children at school or college receiving online learning and 17 had no children receiving online learning. During the data analysis process, the recordings were firstly transcribed and NVivo 11 was used for the coding process. To protect participants’ confidentiality, their personal information was anonymised.

## Findings

During the lockdown, millions of workers in China were forced to shift their working arrangements to WFH. Due to national regulations, their working family members were also likely to shift to WFH. Further, among other COVID-19 specific regulations, schools and colleges were closed and online learning was implemented for students. In this context, ‘home’, substituting for the physical role of offices and schools, was transformed into a multifunctional space in which not only daily family life continued, but also adults engaging in e-working and children receiving e-learning. Around half of the respondents of this research reported their work productivity declined when working from home during the lockdown and the leading reason for such decline was family interference with work.

**Figure 1.**
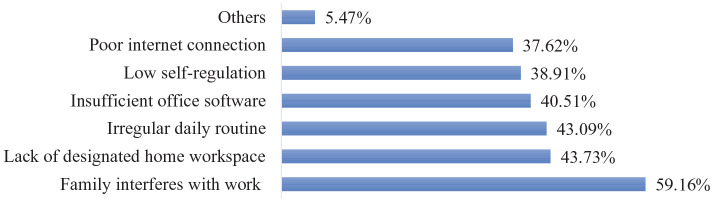
Reasons causing decreased work productivity (n=311). Source: Authors’ compilation based on the survey.

Although compared to actual work productivity, perceived work productivity may be biased by respondent judgement (Bailey and Kurland, 2002), many scholars have asserted that perceived productivity, widely used in WFH research, can be reliable and valid (Baker et al., 2007; Feng and Savani, 2020). When asked whether they perceived their work productivity had increased, or decreased, or maintained during the lockdown, among the 602 remote workers in our survey, only 8.31% (50 respondents) stated their work productivity had increased, 51.66% (311 respondents) reported that their work productivity had decreased, and 40.03% (241 respondents) noted their work productivity maintained the same level as before the lockdown. As demonstrated in Figure 1, among the 311 respondents with decreased work productivity, the interference of family domains with their work domain (59.16%) was the leading reason behind the decline, followed by lack of dedicated home workspace (43.73%), irregular daily routine (43.09%), insufficient office software (40.51%), low self-regulation (38.91%), poor internet connection (37.62%), and other reasons (5.47%). For example, a 34-year-old female interviewee noted:

While working from home during the lockdown, there were so many things disturbing my work, which reduced my work productivity. For example, sometimes, my daughter requested I help with her home-schooling; sometimes, my husband spoke too loudly during e-meetings with his colleagues, which distracted my attention; sometimes, I had to perform domestic chores during online office hours.

This interviewee’s experience indicated that when all aspects of family members’ daily life, along with work and study activities, occurred under one roof, remote workers’ WFH unavoidably interfered with their daily life domain (work-daily life interference), with other family members’ work domain (work-work interference), and with children’s study domain (work-study interference). Therefore, in addition to conventionally discussed work-daily life interference, this section has further analysed work-work interference and work-study interference.

### Work-daily life interference

Work-daily life interference encompassed two main dimensions; interference in the remote worker’s work and daily activities (e.g., leisure, meals), and interference in the remote worker’s work and family life (e.g., childcare). The latter form of interference often caused noticeable inter-role conflict (e.g., employee role vs parental role). Such conflict operated in two directions: the work role interfered with the family life role, or the family life role interfered with the work role. Several studies have pointed out that work interference in family life is more prevalent than family life interference in work (Eagle et al., 1997), as family life boundaries are more permeable than work boundaries (Frone et al., 1992: 723). In our research, a large number of participants also expressed their concerns about work interference in their daily life. Among 602 survey respondents, 7.31% admitted work severely interfered with daily life, 14.95% responded that work strongly interfered with daily life, 38.70% answered that work interfered with daily life to some extent, 30.07% replied that work minimally interfered with daily life, and only 8.97% stated that work did not interfere with daily life. At the same time, 40% stated daily life did not interfere with work.

Further, our interview data indicated that work-daily life interference was bi-directional, involving four main aspects: temporality, physicality, vocality, and digitality. Firstly, temporal interference concerned the inter-role interruptions to schedule and ordering arrangements and work rhythm control. Many remote workers pointed out that an ‘always-on’ culture notably constructed work interference in daily life, especially, the constant need for availability during evenings and weekends. A 52-year-old male interviewee expressed his frustration:

There were lots of online meetings, which often caused conflicts with my family time, as I was expected to be available online all the time. For example, sometimes, while the whole family was having fun during the weekends, I was suddenly requested to attend a meeting, which spoiled my family time. Further, the length of many meetings was unpredictable and often ran over the planned time: some afternoon meetings ran into the evening, which resulted in postponing the family dinner time.

On the other hand, some interviewees highlighted daily life interference with work time, for example, when shopping for groceries at the supermarket, the queuing time became much longer during the COVID-19 period, which interfered with remote workers’ office hours.

Secondly, physicality was examined in terms of workspace. Without a dedicated home workspace, some remote workers had to turn some daily life spaces (e.g., dining rooms or bedrooms) into temporary workspaces, which undoubtedly led to bi-directional interference between work and daily life. For example, a 32-year-old male interviewee noted:

We live in a small flat and no spare room could be used as a home office or study. During the lockdown, I often worked in the bedroom as it is relatively quiet when the door is closed. But sometimes, my wife needed to access the bedroom and that unintentionally disrupted my work. Further, my wife was used to taking a nap after lunch, and she could only sleep on the sofa in the living room, as the bedroom was occupied by me for work. Without a designated workspace, WFH caused inconvenience to the daily routine of my family.

Thirdly, vocality interfered through distraction from people speaking, movement sounds, and the volume of other devices in the household. During remote workers’ online office hours, other family members needed to move quietly, control their voices when speaking, and lower the volume of television and radio to avoid distractions. Further, remote workers often wore headphones during online meetings to prevent family members from interrupting. A 45-year-old female interviewee stated:

When working from home, it is hard to concentrate due to the various sound distractions from home and the neighbourhood, such as TV sounds, conversations between other family members, cars passing by, kids playing outside. Additionally, one of my neighbours was renovating his flat, which was very noisy at times.

Fourthly, digitality encompassed the quality and quantity of digital infrastructure and devices. For some households, with limited internet bandwidth, it was common for remote workers’ other family members to switch off unnecessary daily-life related digital devices such as digital television to avoid excess bandwidth usage. Although the interference worked in two directions, it was socially acceptable for family members to compromise their non-essential daily-life needs for remote workers’ e-working.

Several studies on working from home during COVID-19 have highlighted gender differences; for instance, in the United States (Feng and Savani, 2020) and in Australia (Craig and Churchill, 2021), women encountered a higher degree of work-family interference than men due to housework and childcare. But our data indicated that in China, such gender differences were not significant. Among married respondents, 58.76% of female remote workers experienced work-family interference, compared to 53.73% of male remote workers. Why did Chinese women not experience substantially more work-family interference than men? One of the main reasons was the multigenerational living arrangements in China. Living together, elderly parents assisted their adult children with housework and raising grandchildren (Yu and Xie, 2018: 1068). In China, 58% of parents were assisted with childcare by grandparents, compared to just 10% in the United States and 12% in Europe (Xu, 2021). Relieved of housework and childcare responsibilities, working parents, especially working mothers, were enabled to devote more time to work. For example, a 36-year-old female interviewee noted:

My parents-in-law live with us and they looked after my daughter when I was engaged in online work. In order to prevent my daughter from disturbing me, they often played card games on the balcony, shutting the balcony door.

### Work-work/work-study interference

Due to shifting work and study arrangements during the lockdown, many households had more than one remote worker and/or children engaged in online learning. Hence, it was essential to explore the interference between an individual’s work and family members’ e-working and e-learning (i.e., work-work/work-study interference). The data revealed that family members’ work and study notably interfered with remote workers’ productivity. As demonstrated in Table 1, a higher percentage of respondents experienced decreased work productivity when there were other family members working from home and children studying online. To clarify, among respondents who were the sole WFH member and had no online learning children in the household, 43.94% reported their work productivity decreased. Among respondents living with more family members working from home and without online learning children, 48.24% reported their work productivity decreased. Among respondents as the sole WFH member and with online learning children in the household, 53.66% reported their work productivity decreased, and among respondents living with other family members working from home and with online learning children, 56.88% reported their work productivity decreased.

**Table 1.** Work productivity and family members’ work and study arrangements (n=599).

	Work Productivity Decreased		Work Productivity Maintained		Work Productivity Increased		Total	
	Number	Percent	Number	Percent	Number	Percent	Number	Percent
One member WFH without online learning children	58	43.94%	63	47.73%	11	8.33%	132	100%
Two members or more WFH without online learning children	41	48.24%	39	45.88%	5	5.88%	85	100%
One member WFH with online learning children	88	53.66%	66	40.24%	10	6.10%	164	100%
Two members or more WFH with online learning children	124	56.88%	73	33.49%	21	9.63%	218	100%
Total	311	51.92%	241	40.23%	47	7.85%	599	100%

Source: Authors’ compilation based on the survey.

**Figure 2.**
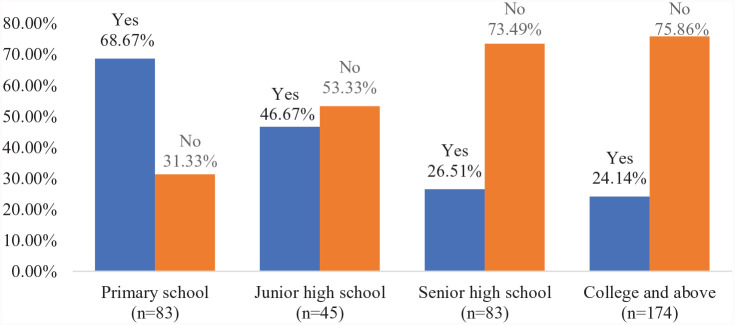
Whether children’s e-learning interferes with remote workers’ e-working (n=385). Source: Authors’ compilation based on the survey.

Children’s e-learning seemed to interfere more with adults’ e-working than the interference from other adult members’ e-working. Further, the interference varied based on student educational level; primary school children’s e-learning interfered the most with parents’ work compared to children in high school and college. As shown in Figure 2, among the remote workers with primary school children, 68.67% stated that online learning interfered with their e-working, compared to 46.67% with junior high school children, 26.51% with senior high school children, and 24.14% with college (and above) students.

In the analysis of work-daily life interference presented in the previous section, four main aspects of interference (temporality, physicality, vocality, digitality) were identified. While examining work-work and work-study interference, these four aspects were in operation. First, temporally, as for work-work interference, family members interrupted each other during their online working hours and such work-work temporal interference was often bi-directional. For example, a 38-year-old male interviewee recounted:

My wife was not so good at the newly introduced office software at the beginning of the lockdown. When she had troubles with software, she often asked me to solve it for her, even though I was working. This happened several times, and each time, I stopped my work immediately to help her. Once, due to the software reinstalling, I spent almost one hour on her laptop, which surely affected my work. But sometimes, I also interrupted her work.

As for work-study temporal interference, it was common to find parents’ office hours considerably interfered with students’ e-learning. For one, children, especially those in primary school, needed parental assistance with ICT (e.g., logging into an online classroom, ensuring optimal sound and video effects, submitting homework online). Moreover, parents worried about leaving children alone for hours, potentially distracting themselves due to lack of self-discipline, for instance, browsing websites or playing computer games. Thus, parents were often present to provide assistance before each online class and afterwards, making sure everything went smoothly. Even when parents sought to focus on their own work, they frequently came back to check in on the progress of children’s e-learning. Table 2 shows that among the 385 respondents with children receiving online learning, 40.22% kept an eye on their children during e-learning hours. The lower the educational level of the children, the higher the chances that parents were present. For households with primary school children, 78.32% of working parents were present, with 51.11% for junior high school students, 39.76% for senior high school students, and 13.79% for college (and above) students.

**Table 2.** Whether parents were present during children’s online learning (n=385).

	Yes		No		Total	
	Number	Percentage	Number	Percentage	Number	Percentage
Primary school	65	78.32%	18	21.68%	83	100%
Junior high school	23	51.11%	22	48.89%	45	100%
Senior high school	33	39.76%	50	60.24%	83	100%
College and above	24	13.79%	150	86.21%	174	100%
Total	145	40.22%	240	59.78%	385	100%

Source: Authors’ compilation based on the survey.

Secondly, as most households lived in moderately sized flats within high-rise buildings, they often physically lacked dedicated home workspace or study space. The national per capita floor space averaged 29m^2^ in urban China but major cities (e.g., Beijing, Shanghai) were below this national average (Yi and Huang, 2014). Therefore, when someone was working from home while other family members needed space to work or study online, unavoidable conflicts resulted. The work-work or work-study physical interference was often bi-directional, but when encountering work-study interference, most parents put children’s e-learning as the priority, compromising their workspaces for children’s study. For example, a 41-year-old female interviewee noted:

When my son has online classes, my husband and I always leave the best spot in our flat to him, where the light is sufficient and it is quiet too. After my son finished his classes, either my husband or I moved to that spot for working.

This practice pointed to the asymmetry of the work-study interference; children’s study interfered much more with parents’ work than the other way around, as parents could often arrange their work more flexibly.

Thirdly, when working from home, workers often needed to conduct video/voice calls with colleagues or clients. During online learning, students were encouraged to participate in classes verbally. Hence, family members disturbed each other’s work or study, and such work-work/work-study vocal interference worked in two directions. For example, a remote worker’s Zoom meeting disturbed the work of other family members and children’s study, while children’s class discussions distracted adults’ work in turn. To reduce such vocal interference, several interviewees noted that they had to control their voices when talking online or via telephone.

Fourthly, inadequate digital infrastructure and devices caused interference between remote workers’ work and family members’ work and study. For example, some households’ internet bandwidth was not sufficient for multiple family members’ e-working and e-learning; therefore, their online activities invisibly competed for bandwidth. Such work-work/work-study interference was bi-directional. Further, a large number of students did not have their own online learning devices and often needed to use their parents’ devices, which inevitably affected parents’ work. Several interviewees mentioned that there was only one computer at home, which was necessary for their WFH as well as for their children’s study needs. Commonly, parents let children have priority in using the computer.

## Discussion

The above section reveals during the lockdown, remote workers not only encountered work-daily life interference but also work-work interference and work-study interference. These three dimensions of interference are bi-directional: remote workers’ e-working can be interrupted by daily-life, family members’ e-working, and children’s e-learning, and vice versa. Further, each dimension of interference includes four main aspects (i.e., temporality, physicality, vocality, and digitality). A mixture of these heterogeneous dimensions and aspects of work-family interference influences remote workers working from home, which is coined as ‘assemblages of work-family interference’. The term ‘assemblage’ is employed for two reasons. Existing literature on ‘assemblage’ highlights its status as a mixture of heterogeneous elements (Duff and Sumartojo, 2017; Latour, 2005); further, it implies a dynamic process, rather than being static (Baker and McGuirk, 2017; Wise, 2005).

The empirical data indicate the mixture of work-family interference largely depends on the family members’ work and study arrangements. As shown in Table 3, there are four possible permutations: I) if only one member is working from home without online learning children, this remote worker likely encounters primarily work-daily life interference; II) if two members or more are working from home without online learning children, the remote workers encounter not only work-daily life interference but also work-work interference; III) if one member is working from home and there are online learning children at home, this remote worker encounters both work-daily life and work-study interference; and IV) if two members or more are working from home with online learning children at home, remote workers encounter all three types of interference (work-daily life, work-work, work-study). Further, as each dimension may include four aspects of interference, a type IV worker is likely to experience an assemblage of 12 forms of interference (i.e., temporal, physical, vocal, and digital work-daily life interference, temporal, physical, vocal, and digital work-work interference, temporal, physical, vocal, and digital work-study interference). In contrast, a type I individual, the sole remote worker in a household and without online learning children, may only encounter an assemblage of four forms of interference (i.e., temporal, physical, vocal, and digital work-daily life interference).

**Table 3.** Assemblages of work-family interference while working from home.

Dimension	Aspect	One member WFH without online learning children	Two members or more WFH without online learning children	One member WFH with online learning children	Two members or more WFH with online learning children
Work-Daily Life	Temporal	√	√	√	√
	Physical	√	√	√	√
	Vocal	√	√	√	√
	Digital	√	√	√	√
Work-Work	Temporal		√		√
	Physical		√		√
	Vocal		√		√
	Digital		√		√
Work-Study	Temporal			√	√
	Physical			√	√
	Vocal			√	√
	Digital			√	√

Source: Authors’ compilation.

Further, the work-family interference a remote worker encounters varies over time. Consider the example of work-study interference: during e-learning hours, remote workers may need to let children use their computer for online learning, representing digital interference; further, the child may need technological assistance throughout the e-class, interrupting a remote worker’s official working hours (temporality). However, the remote worker is unlikely to encounter this identical assemblage of work-family interference during children’s non-school hours.

To avoid work-family interference, scholars emphasise the importance of boundary segmentation among different domains when working from home (Derks et al., 2016; Kossek et al., 2006). Our findings reveal that due to the assemblages of work-family interference, domain boundaries become multifaceted (i.e., the boundary between work and daily life, between work and work, and between work and study). Further, transboundary interaction and cross-domain interpenetration engage in a dynamic process. Thus, boundaries are shaped and reshaped along with the change of assemblage, which causes more challenges to effective boundary segmentation. Instead, the boundaries become more blurred and gradually dissolve, for several reasons.

First, as regards the housing and living patterns in major Chinese cities, many households live in flats with limited living space. During the lockdown, with all family members’ activities (e.g., living, working, studying) carried out under one roof, the boundaries between work and other domains are easily permeable, which causes work-family interference, especially for individuals without a dedicated workspace at home. Second, workers in China are influenced by Confucian values such as hard work, endurance, and collectivism, among others (Cho and Choi, 2018). Due to the pandemic, it is estimated that 92 million Chinese workers became unemployed in 2020 (Zhang, 2021). For those who had retained their job positions, their work ethic and social expectations drove them to work extra hard (e.g., long work hours) and to devote less attention to the family, which, in turn, unintentionally causes more work-family conflicts and interference. Several interviewees explicitly state that in order to overcome work and family inter-role conflict, they had to work in the evening. Third, as a middle-income country, many households in China cannot afford for each member to possess a separate digital device, causing sharing conflicts. According to National Bureau of Statistics (2020), the number of computers per one hundred households in China was 53.2 in 2019. In other words, around half of Chinese households do not even have a computer. They often have to borrow one from relatives or friends for essential use. Further, digital infrastructure such as broadband connections for e-working and e-learning is yet to be improved in China, especially in small and middle cities and rural areas.

## Conclusions

It is estimated there were around 400 million Chinese people working from home during the national lockdown in 2020 (Mobtech, 2020). As one of the few studies examining the phenomenon of WFH in China, this article has revealed the following key empirical findings based on a survey of 602 respondents and 36 in-depth interviews with Hubei urban residents who worked from home during the lockdown. Firstly, 51.92% of survey respondents report their work productivity decreased compared to pre-COVID times, only 7.85% believe their work productivity increased, and 40.23% note their work productivity was the same as before. Secondly, among workers with decreased productivity, family interference with the work domain is the leading cause of the decline. Thirdly, as family members’ work and study arrangements had also shifted online during the lockdown, it is common to find that within one household, multiple family members must juggle e-working or/and e-learning, which unavoidably interfere with each other.

To provide a comprehensive understanding of WFH during the lockdown, an analytical framework of ‘assemblages of work-family interference’ has been proposed. Remote workers encounter a mixture of heterogeneous forms of work-family interference, including three dimensions (i.e., work-daily life, work-work, work-study) and four distinct aspects (i.e., temporality, physicality, vocality, digitality). These interferences can be bi-directional: remote workers’ e-working can be interrupted by daily-life, family members’ e-working, and children’s e-learning, and conversely, their e-working can disturb daily-life, family members’ e-working, and children’s e-learning. Further, the mixture of dimensions and aspects of interference is not always consistent; the assemblage of work-family interference varies over time. Boundary segmentation among different domains is vital for remote workers (Derks et al., 2016; Kossek et al., 2006). Through assemblages of work-family interference, remote workers face challenges to segmenting boundaries effectively, as boundaries become multifaceted and the forms of interference are not static.

According to China Internet Network Information Center (2021), COVID-19 accelerated WFH in China in the long term, especially for knowledge workers. A few policy recommendations are formulated. First, work-life balance should be better promoted. ‘Always-on’ culture and overtime working patterns can be tackled in China through the ‘right to disconnect’, as advocated in OECD countries (Organisation for Economic Co-operation and Development (OECD), 2020). Furthermore, the All China Federation of Trade Unions (ACFTU) should be more active in addressing issues emerging from the phenomenon of WFH. Second, low-income households should be provided with ICT equipment (e.g., computers, laptops) to meet their demand for e-working and online learning. For example, during COVID-19, the UK government provided more than 1.3 million ICT devices to help disadvantaged students (Govuk, 2021). Moreover, digital infrastructure (e.g., fast and reliable broadband) should be accessible across China in both urban and rural areas. Third, an allowance for home workplaces should be considered, which can be used to compensate increasing energy bills and/or to improve the working environment at home (e.g., soundproofing insulation).

In sum, this research contributes to empirical understandings, conceptual debates, and policy implications in the field of WFH. However, one of the limitations to this study is worth noting: the informants for this study all come from the urban context. Among the 1.4 billion population in China, around 60% (840 million) lived in cities and 40% (560 million) lived in rural areas in 2019 (National Bureau of Statistics, 2020). Due to the poor digital infrastructure in rural China and the low levels of ICT equipment among rural households, concern over this rural-urban digital divide has been raised (Song et al., 2020). For example, the number of computers per one hundred households is only 27.5 in rural China, compared to 72.2 in cities (National Bureau of Statistics, 2020). Hence, future empirical studies in WFH should pay more attention to residents in rural China.
